# Is TRPA1 *Burning Down* TRPV1 as Druggable Target for the Treatment of Chronic Pain?

**DOI:** 10.3390/ijms20122906

**Published:** 2019-06-14

**Authors:** Simona Giorgi, Magdalena Nikolaeva-Koleva, David Alarcón-Alarcón, Laura Butrón, Sara González-Rodríguez

**Affiliations:** 1 Instituto de Investigación, Desarrollo e Innovación en Biotecnología Sanitaria de Elche (IDiBE), Avda de la Univesidad s/n, Universidad Miguel Hernández, 03202 Elche, Spain; sgiorgi@umh.es (S.G.); mnikolaeva@umh.es (M.N.-K.); david.alarcon.alarcon.91@gmail.com (D.A.-A.); lbutron@umh.es (L.B.); 2 AntalGenics, SL. Ed. Quorum III, Parque Científico Universidad Miguel Hernández, Avda de la Universidad s/n, 03202 Elche, Spain

**Keywords:** TRPA1, pain, inflammation, neuropathy, molecular modulation

## Abstract

Over the last decades, a great array of molecular mediators have been identified as potential targets for the treatment of chronic pain. Among these mediators, transient receptor potential (TRP) channel superfamily members have been thoroughly studied. Namely, the nonselective cationic channel, transient receptor potential ankyrin subtype 1 (TRPA1), has been described as a chemical nocisensor involved in noxious cold and mechanical sensation and as rivalling TRPV1, which traditionally has been considered as the most important TRP channel involved in nociceptive transduction. However, few TRPA1-related drugs have succeeded in clinical trials. In the present review, we attempt to discuss the latest data on the topic and future directions for pharmacological intervention.

## 1. Introduction

The International Association for the Study of Pain (IASP) defines pain as “an unpleasant sensory and emotional experience associated with actual or potential tissue damage, or described in terms of such damage”. This sensory response is necessary for survival since it warns us about potential injuries; however, what are the consequences if this response fails or becomes chronic? Sometimes the pathways that should be only activated in presence of harmful stimuli lose their physiological function and lead to the development of chronic painful pathologies. This kind of disorders have an important societal impact in health and in economic terms, affecting 740 million people in Europe where ~20% of the population suffers from chronic pain [1]. As a consequence, this prevalence results in a large number of medical consultations, becoming an important economic investment [2]. In addition, these pathologies cause absenteeism from work, being responsible for 500 million sick days in Europe and costing €240 billion per year [1]. Chronic pain is the main reason why people leave their employments prematurely [3]. Furthermore, the long-term consumption of current available treatments such as NSAIDs (non-steroidal anti-inflammatory drugs) or opioids induces detrimental side effects limiting their use. Accordingly, understanding how pain pathways work is still an overriding goal in order to discover new molecular targets as well as to improve already available pharmacological treatments.

Somatosensations such as touch, proprioception, nociception, or thermosensation are detected by the somatosensory system where primary sensory neurons are responsible for receiving the aforementioned stimuli. In terms of pain, sensory neurons can be divided into two main types: nociceptive and non-nociceptive. The latter detects stimuli in a continuous range of intensity, while the nociceptive sensory neurons show a higher activation threshold being able to detect potentially harmful stimuli. The process by which primary sensory neurons detect and transmit nociceptive information is denominated nociception and the neurons responsible for this action are nociceptors [4,5].

Nociceptors soma is located either in trigeminal (TG) or dorsal root ganglia (DRG); nerve fibres depart from these ganglia that connect to somatic, visceral, and trigeminal regions as well as to the spinal cord and brain transmitting the nociceptive signals between peripheral (PNS) and central (CNS) nervous system [5]. The most widely used classification of nociceptors is based on the axon type displayed: (i) Aβ fibres that, with high conduction, detect innocuous stimuli; (ii) Aδ fibres that, with lower conduction velocities, detect mechanical and thermal stimuli and are responsible for acute pain sensation; and (iii) C fibres that are activated by chemical, mechanical, and thermal stimuli [4,6].

Pain propagation is usually described in four phases: transduction, transmission, modulation, and perception. Briefly, peripheral nerve fibres, which innervate skin, muscles, and viscera detect and translate stimuli into action potentials that travel along the axon, which penetrates into the dorsal horn of the spinal cord. This nociceptive information eventually reaches supra-spinal areas in the brain where is integrated [7].

In pathological conditions, these pathways might be altered and nociceptors undergo a sensitisation process. In such case, their activation threshold decreases, leading to an increase in sensitivity and excitability of the nerve fibres. During healthy conditions, when the stimulus ceases, the nociceptive system reaches again its basal state, recovering its activation threshold, which takes no place in pathological conditions. This process can occur both peripherally and centrally [8,9].

Nociceptors express a broad range of ion channels and/or receptors that have to be considered in order to understand the molecular pathways they take part in. Among these mediators, TRP channels are one of the most prominent family studied in the field of pain [10], and since TRPA1 seems to play a key role in nociception, it will be discussed in the present review.

## 2. TRP Channels Family

The TRP channels superfamily was initially described by the discovery of the *Drosophila*–TRP channels in genetically altered photoreceptors. This mutation showed transient voltage responses to the light stimuli; thus, it was denominated transient receptor potential [11,12]. These ion channels form a family of evolutionarily conserved ligand-gated ion channels that act as sensors of physical and chemical stimuli. Unlike other channels, this family possesses a wide range of activators (chemical compounds, temperature, mechanical stimuli, osmolarity, lipids, light, oxidative species, and pH), and their regulation (transcription, alternative splicing, glycosylation, and phosphorylation) is linked to an extensive tissue distribution and thus to different biological roles [13]. They are nonselective cationic channels that permeate mainly calcium but also potassium and sodium. The influx of calcium to the cell triggers several actions such as cellular proliferation, cell death, gene transcription, and the release of neurotransmitters.

To date, there have been several TRP channels described that are present in mammals as six families: TRPC, TRPM, TRPV, TRPA, TRPP, and TRPML based on amino acid homologies (IUPHAR), being TRPV1-4, TRPA1, and TRPM8 the most prominent expressed in nociceptors and the most extensively studied [13]. Additionally, TRPV1, TRPA1, and TRPM3 conform an important thermoregulatory system in sensory neurons [14].

### 2.1. TRPV1 Channel

In the description of the nociceptive transmission, TRPV1 was the first TRP cloned channel in 1997 and it is a cationic channel that displays high calcium permeability [15]. TRPV1 is found in both peripheral and central terminals of nociceptors in the DRG, TG, nodose ganglion (NG), geniculate ganglion (GG), and jugular ganglion [16,17,18,19]. TRPV1 also modulates pain transmission at the first sensory synapse [19,20]. This channel acts as a polymodal sensor since it can be activated by noxious heat (≥43 °C), low pH (<6.5), and some other exogenous or endogenous compounds (camphor, allicin, toxins, anandamide) besides the well-known agonist, capsaicin.

It is estimated that approximately 35–50% of all DRG or TG neurons are TRPV1 positive [21], being a large population of unmyelinated C-fibres and a small population of thinly myelinated Aδ fibres. TRPV1 expression is usually accompanied by the expression of some other pain mediators such as Substance P (SP) or Calcitonin Gene-Related Peptide (CGRP).

Regarding pain involvement, it has been described that the administration of some of the aforementioned chemicals evokes pain-related behaviours, and they are mediated by TRPV1 as TRPV1-deficient mice showed. These animals displayed a full loss of behavioural responses to capsaicin and reduction in heat responses. Intriguingly, some studies also showed that TRPV1 null mice display normal sensitivity to acute noxious heat [22,23]. However, normal responses to mechanical stimuli were observed. In vitro data revealed that calcium influx or electrophysiological responses to capsaicin are abolished in these animals that might suggest that other mediators could participate in the sensation of noxious heat when TRPV1 is not expressed [24,25].

### 2.2. TRPA1 Channel

Originally denominated ANKTM1 (ankyrin-like with transmembrane domains protein 1) but currently called transient receptor potential ankyrin 1 (TRPA1), this receptor has been conserved in different species during evolution. It was initially identified in human foetal lung fibroblasts as a transformation-associated gene product [26]. It is a non-selective cationic channel and it is expressed in the PNS and CNS. TRPA1 is clearly linked to pain due to its expression in nociceptive structures [7,10,27,28,29]. It is generally co-expressed with TRPV1 in nociceptors and also acts as a polymodal sensor as it might be activated by several chemical, thermal (≤18 °C), mechanical, and osmotic stimuli [30].

Since different stimuli can activate TRPA1, each mode of activation indicates a different role for this channel in a different pain pathway. Thus, in chemosensation, TRPA1 can be activated by a plethora of molecules which are able to evoke a stinging sensation [31,32]. An important group of TRPA1 activators are reactive oxygen species (ROS) that together with ultraviolet light can stimulate TRPA1 [33,34]. Indeed, higher levels of ROS are usually found during inflammation [35]. Likewise, TRPA1 is a thermosensor as it has been shown that cold can elicit TRPA1 currents in in-vitro systems [36]. However, this stimulation is minimal compared to chemical agonist-induced currents, which means that cooling enhances TRPA1 currents in presence of TRPA1 agonists [37]. Furthermore, cold induces the release of ROS or calcium, which in turn activates the TRPA1 channel [35]. Finally, TRPA1 also participates in mechanosensation but it is not intrinsically mechanosensitive as shown in null mice after high pressure stimuli application [38].

**Figure 1:**
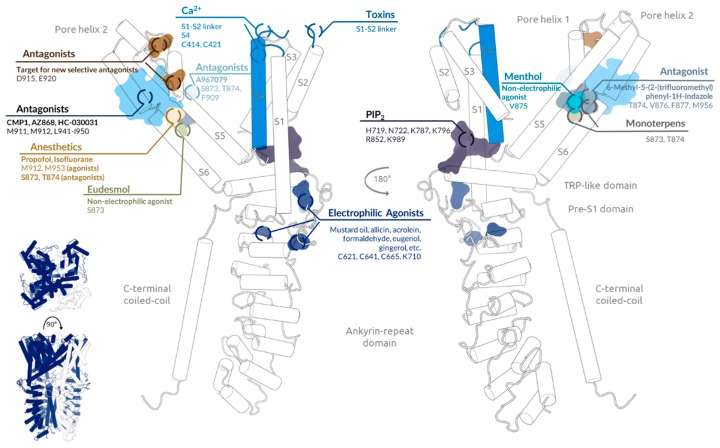
Main TRPA1 chemical modulators binding sites. General view of human transient receptor potential ankyrin subtype 1 (TRPA1) structure is shown in the inset. Detailed view of one subunit (white) is shown on the right. Main chemical modulators’ binding sites are highlighted on the structure. Labels tag key residues for each site, give an example of, and/or classify each substance.

From a structural point of view, understanding TRPA1 function and regulation is tightly linked to unveiling the structural mechanisms responsible for them. Recently, the three-dimensional atomic structure of the full-length human TRPA1 channel has been determined with ~4 Å resolution [39] (PDB Code: 3J9P). As the rest of the TRP superfamily [40], TRPA1 forms a homotetramer. Each monomer has an intracellular N-terminal ankyrin repeats domain and a C-terminal coiled-coil domain. The N-terminus is followed by a linker domain and a Pre-S1 helix. Four transmembrane helixes come after (S1–S4), a S4–S5 linker, S5, and S6. Between these last, the pore helix 1 and 2 are found. Finally, the TRP-like domain connects with a β-sheet that ends into the C-terminus. The oligomer structure reveals channel organisation, pore architecture, and key regulatory interactions and establishes a base for structure-based design of analgesic, anti-inflammatory, and anaesthetic agents (Figure 1, Table 1).

Many irritant chemicals are potent electrophiles that activate the channel through covalent modification of cysteine (C621, C641, C665) or lysine (K710) residues on its N-terminus [41,42], specifically the Pre-S1 region. These agonists include mustard oil, tetrahydrocannabinol, allicin, acrolein, formaldehyde, N-methylmaleimide [43,44,45], cinnamaldehyde [46], eugenol, gingerol, and thymol [47]. TRPA1 is also activated by Ca^2+^ ions [48,49] binding to residues C414 and C421 in the ankyrin-repeat domain [50]. Nonetheless, recently it has been suggested that Ca^2+^ could also bind to negatively charged amino acids in the S1–S2 linker [51], a region that is also involved in a tarantula toxin binding [52], but also to residues in the S4 segment [53]. Menthol is an exception given that it activates the channel by binding to residues from the S5–S6 transmembrane domain especially to V875 [54] like other non-electrophilic agonists such as eudesmol (S873) [55] and protons [56]. 6-Methyl-5-(2-(trifluoromethyl)phenyl)-1H-indazole has been identified as an inhibitor that acts directly on the predicted menthol binding-site located on the outer-side of the pore domain binding to residues T874, V876, F877, and M956 [57]. Several studies point out the pore domain as a druggable binding site of TRPA1. Some antagonists such as CMP1 [58], AZ868 [59], and HC-030031 [60] bind to residues M911 and M912 from the inner mouth of the selectivity filter and/or a group of residues in S6 from I940 to I950 [61]. It has been determined that the antagonist A-967079 [62] binds to a discrete site different from the one of HC-030031 formed by residues S873, T874, and F909 [39]. The inhibitory effects of monoterpens also require S873, T874 [63]. Some general anaesthetics like propofol and isofluorane share this binding pocket but they also interact with M912 and M953 required for their agonistic effects [64]. It is also suggested that the region of the selectivity filter (D915) along with the residue E920 located on the outer mouth of the pore which attracts cations to the pore could conform a binding site for TRPA1 selective antagonists in terms of highly charged molecules [65]. Another notable structural regulatory region is the one beneath the lower segment of the S1–S4 sensor domain capable of binding to phosphoinositides and regulating channel gating. It has been determined that the negatively charged inositol triphosphate head group of PIP_2_ contacts residues H719, N722, K787, K796, R852, and K989 [66].

The pore phenomenon where TRPA1 [67,68], like other TRPs, is found on its dilated state is of great interest because it could stand for a novel way for delivering hydrophilic or low-permeability compounds, lower drug doses required for therapeutic effect, thus decreasing the risks of side effects [69] like in the case of capsaicin-gabapentin application on TRPV1 [70]. Moreover, during the dilation process, new potentially therapeutic binding sites could be revealed [71].

As pain signalling relies on sensitization of nociception-related TRPs, another noteworthy strategy to control pain relies on the inhibition of only sensitized TRP channels [72]. Between all factors involved in sensitization, the development of agents that specifically inhibit post-translational changes such as phosphorylation or glycosylation or blocking the translocation process of the channel from the cytoplasm to the cell membrane [73,74,75] appear as a more realistic intervention and reduce possible undesirable side effects. However, the exact molecular basis for this process for TRPA1, in which PKA and PLC seem to be involved [37,76], is still unclear.

## 3. TRPA1 in Inflammatory Pain

### 3.1. The Double Face of TRPA1 in Gastrointestinal Pain Models

TRPA1 channel has a pivotal role in mechanosensation and nociception within the viscera [77]. It is highly expressed in human and rat enterochromaffin cells which store serotonin and can be stimulated by TRPA1 agonists [78,79]. Interestingly, serotonin participated in the pathogenesis of post-infectious irritable bowel syndrome and modulated visceral nociception, being the levels of serotonin in patients higher than in healthy controls [80], suggesting that TRPA1 is an interesting target in the treatment of this syndrome.

TRPA1 has also been investigated in inflamed colon models, where nerve growth factor (NGF) and glial derived neurotrophic factor (GDNF) are increased potentiating expression and functionality of TRPA1 and TRPV1 in skin, muscle, and colon [81]. TRPV1 and TRPA1 concert has been further investigated in acute colitis and visceral hypersensitivity model, where the blockade of both channels reduced visceromotor responses when compared to a single channel inhibition. Consistently, colitis symptoms might be alleviated by intrathecal administration of TRPA1 antisense oligodeoxynucleotide or by pharmacological inhibition of TRPA1 [82,83,84]. Similarly, colitis in mice was attenuated by capsazepine due to a desensitisation of TRPA1 [85] or to the reduction of neuropeptides release such as SP and CGRP [86].

On the contrary, selective TRPA1 agonists relieved colitis and abdominal pain in murine models [87,88]. This effect is still unclear, however, the inhibition of TRPA1 did not improve in a different model of colitis in mice [89], highlighting the importance of the experimental model when studying the TRPA1 channel role in irritable bowel syndrome. Additionally, a protective role for TRPA1 has been postulated in intestinal mucositis and in fibrotic inflammatory disorders such as Chron’s disease [90,91].

### 3.2. TRPA1 Is Involved in Joint and Muscle Pain

In human chondrocytes, TRPA1 is functionally expressed during osteoarthritis (OA) and it participated in mechanical hypersensitivity and inflammation in OA animals, and its expression was increased after treatment with inflammatory factors [92,93]. Complete Freund’s adjuvant (CFA) or monosodium iodoacetate (MIA) models showed that the blockage of TRPA1 relieved some of the painful symptoms [94,95,96].

TRPs are also involved and upregulated in rheumatoid arthritis (RA) [97] where the preincubation of synovial fibroblasts with TNF upregulated and sensitized TRPA1, reducing cell viability by inducing necrosis [98]. In human and murine synovial fibroblasts, the activation of TRPV1 and TRPA1 by endocannabinoids downregulated proinflammatory cytokines, and the treatment with endocannabinoids alleviated collagen-induced arthritis in mice [99].

Gout is another joint disease in which monosodium urate (MSU) crystals deposit intra-articularly and cause painful arthritis [100]. In mice, painful symptoms of intra-articularly injected MSU crystals might be relieved when TRPA1 is inhibited by antagonists or genetically ablated [101]. The joint could also be affected by yeast *Candida osteomyelitis* that induces pain and bone destruction, in which TRPV1 and TRPA1 deficiency caused osteoinflammation and diminished CGRP production [102].

Intramuscular injections of TRPA1 agonists aroused nocifensive responses and mechanical hyperalgesia in muscle afferents [103], and in a model of masseter inflammation, TRPA1 mRNA expression was found to be increased in the TG [104,105]. Inhibition of both TRPA1 and TRPV1 in masseter muscle decreased spontaneous pain but did not alleviate bite-evoked pain [106]. Consistently, in orofacial pain models, intramuscular injection of AP18, a selective TRPA1 antagonist, blocked the progress of acute mechanical hypersensitivity and persistent muscle pain [103]. Additionally, in a model of skin and deep tissue incision, TRPA1 pharmacological blockade reduced spontaneous guarding pain behaviour. Interestingly, oxidative TRPA1 agonists (ROS and H_2_O_2_) were increased in incised skin and muscle [107]. Finally, it has been more recently hypothesised that TRPA1 may be beneficial in delaying the progression of Duchenne’s muscular dystrophy as tetrahydrocannabidivarin showed improving myotube formation through the activation of TRPA1 [108].

### 3.3. TRPA1 and TRPV1 Cooperate in Skin Pathologies

In mouse models of pruritus and psoriasis, genetic ablation of TRPA1 abrogated scratching and improved skin lesions, demonstrating that the channel controls itch transduction to the central nervous system and pathophysiological alterations in the skin [109,110]. TRPV1 role in skin diseases has also been investigated, showing that both channels are involved in IL-31 induced itch; indeed, TRPV1 or TRPA1 pharmacological antagonism and ROS scavengers decreased itch in mice [111,112].

In allergic contact dermatitis (ACD) models, it is not clear yet whether TRPA1 and/or TRPV1 are implicated in the pathophysiology. Genetic ablation or pharmacological blockage of TRPA1, but not TRPV1, decreased ACD typical symptoms and histamine independent scratching behaviour [113]. Notably, oxidative stress-induced itch is mediated by TRPA1 and is TRPV1-independent, while chloroquine and BAM8-22 induced TRPA1-dependent scratching behaviour that is histamine-independent [114,115]. Interestingly, chloroquine activated the itch-related G-protein-coupled receptor MrgprA3 to trigger histamine-independent itch, and TRPA1 has been found to signal downstream of MrgprA3 [115]. Another hypothesis about the interaction between TRPA1 and TRPV1 in ACD has been recently published demonstrating that both channels are required for the development of ACD but only TRPV1 protected from skin inflammation [116].

Expression of TRPA1 in dermal sensory nerves during atopic dermatitis (AD) was markedly elevated in injured skin biopsies from AD patients when compared to healthy controls. Thus, TRPA1 is not only necessary as a sensor for pruritogens but is also essential in maintaining skin inflammation [109,113,117].

### 3.4. TRPA1 Is a Sentinel for External Threats in the Airways and Urinary Tract

TRPA1 is expressed in the airways where it functions as a nocisensor for external threats [118]. Indeed, stimulation of C-fibres in the airways caused the release of inflammatory neuropeptides (CGRP and SP) that induce neurogenic inflammation. Extended and prolonged inflammation can lead to cough, asthma, and chronic obstructive pulmonary disease (COPD) and, interestingly, TRPA1 expression has been demonstrated in immune cells involved in the inflammatory response in asthma and COPD [119,120]. Unfortunately, to date, TRPA1 role has not been investigated in experimental models of COPD.

Several inflammatory compounds such as nitric oxide, protons, and ozone activated human TRPA1 heterologously through an oxidative mechanism [121,122], highlighting the importance of oxidative stress and TRPA1 in inflammatory conditions. Consistently, exposition to cigarette smoke may increment extracellular ROS, which activate TRPA1 inducing an increase of intracellular ROS and activation of pro-inflammatory signalling [123].

Another respiratory clinical condition is allergic rhinitis. In-vitro, periodic applications of antihistamine azelastine hydrochloride and/or corticosteroid fluticasone propionate desensitized sensory neurons expressing TRPA1 and TRPV1 [124]. The two channels have also shown a synergistic effect in rat vagal pulmonary sensory neurons and in the apnoeic response to application of AITC or capsaicin [125,126]. Moreover, TRPV1, TRPA1, and TRPM8 agonists produced nasal pain and smart in healthy volunteers and capsaicin and mustard oil also caused rhinorrhea [127]. TRPA1 is also expressed in deep airways, specifically in the epithelium facing the bronchial lumina of cystic fibrosis patients where inhibition of the channel led to a decrease of various proinflammatory cytokines [128].

TRPA1-expressing C-fibres comprise 50% of all bladder-innervating sensory neurons and mostly express CGRP, SP, and TRPV1 [129]. The expression of TRPA1 mRNA and protein in both mucosa and DRGs is increased in cyclophosphamide-induced cystitis and can be decreased by treatment with TRPA1 antagonists [130,131,132]. Similarly, spinal cord injury also affected the bladder and the urinary system upregulating TRPA1 protein and mRNA in the periphery but not in the central nervous system [133]. In addition, it has been shown that after intravesical lipopolysaccharide-administration, TRPA1 is implicated in bladder mechanosensory and nociceptive hypersensitivity that also present inflammation, while it was not involved in physiological bladder function [134], suggesting that the channel plays a role in detecting urinary pathogens. Moreover, it has been demonstrated that ROS are involved in urinary bladder disorders, and in a H_2_O_2_-induced cystitis model, TRPA1 contributed to acute bladder hyperactivity but did not seem to play a pivotal role in the pathological development of chronic cystitis [135]. Another frequently used bladder hypersensitivity model that consists on formalin injection increases TRPA1 expression in the bladder mucosa together with NGF and P_2_X_2_ receptors [136]. Formalin is, indeed, a standard substance that causes pain and its effects seem to be usually mediated by TRPA1 [137,138]

### 3.5. TRPA1 Senses Oxidation in the Cardiovascular System

TRP channels are widely expressed in the cardiovascular system with TRPA1 expressed in smooth muscle and endothelial cells of different species [139], making this channel important in the regulation of vascular tone and in the development of atherosclerotic disease accompanied of angina and leg pain [117]. In atherosclerosis, TRPA1 may be an important regulator because of its activation by oxidized low-density lipoproteins, confirming the channel as a sensor of oxidative stress [140,141]. The blockage of the receptor could counteract inflammatory pain in cardiovascular system [142]. Furthermore, TRPA1 is increased in heart after doxorubicin treatment and in human and mouse hypertrophic hearts, where blocking the channel improved these pathological conditions [143,144].

### 3.6. TRPA1 Role in Eye Diseases

TRP channels are found in the eye being TRPV1 the most characterised TRP in this organ because of its role in maintaining homeostasis [145]. TRPV1, TRPA1, and TRPM8 channels appeared to be important in UV corneal sensitisation [146] while TRPV1 and TRPA1 inhibition after alkali burn decreased corneal fibrosis inflammation and opacification [147,148]. TRPA1 was also important for the sensitisation of ocular-responsive trigeminal brainstem neurons in a model for tear-deficient dry eye [149], but further studies are necessary to assess the role of this channel in different eye pathological conditions.

## 4. TRPA1 in Neuropathic Pain

### 4.1. TRPA1 Is Essential for Nerve Injury and Chemotherapy-Induced Neuropathic Pain

Many neuropathic pain models induced by nerve injury are characterised by mechanical and cold hypersensitivity [150,151], suggesting a relevant role for TRPA1 [92,152] as it is a key transducing channel in mechanical/cold-sensing nociceptors [38,153]. Indeed, TRPA1 blockers or genetic ablation decreased nociceptive changes observed in animal models of neuropathic pain [60,154,155,156]. Another standard model of neuropathic pain is the one induced by the administration of chemotherapy drugs that may produce, as side effects, intense cold and mechanical hyperalgesia and/or allodynia, limiting administration to patients [157,158]. Similar to nerve injury models, the blockage of TRPA1 counteracted pain in several models of chemotherapy-induced neuropathic pain [159,160]. Oxaliplatin is often used to establish experimental neuropathic pain, through TRPA1 [161,162] but not TRPV1 sensitisation [163]. It has been shown that aluminium accumulates in DRGs of patients treated with oxaliplatin, increasing cold but not heat hypersensitivity, suggesting not TRPV1 but TRPA1 activation instead [164,165].

### 4.2. TRPA1 Is Upregulated in Neuropathic Pain

Nerve lesion and chemotherapy models of neuropathic pain showed that TRPA1 mRNA and protein are upregulated in peripheral and central terminals of nociceptors and in central sensory pathways [154,166,167,168]. Importantly, TRPA1 upregulation can be accompanied by an increased expression of TRPV1, α-CGRP, SP and pro-inflammatory cytokines release in dorsal root and trigeminal ganglia, cervical spinal cord, and medulla in mice [154,166]. The importance of TRPA1 upregulation has been assessed by studies which showed that blocking TRPA1 upregulation ameliorated pain, for example, microRNA 449a improved neuropathic painful symptoms by decreasing TRPA1 expression in mice [169]. Notably, it has been reported that some models of neuropathic pain undergo upregulation of TRPA1 but not TRPV1. These last studies are characterized by an increase in mechanical and cold, but not heat, sensitivity [164,170]. An important physiological role of TRPA1 is to promote α-CGRP and SP exocytosis as elicitors of vasodilation and neuro-inflammation [171], as seen in a trigeminal neuropathy in which blocking TRPA1 showed decreased levels of α-CGRP, SP, and even TRPV1 gene expression [154].

### 4.3. TRPA1 Modulation in Neuropathic Pain Shares Convergent Signalling Pathways in Different Models of Neuropathic Pain

Recent studies have enlightened some of the mechanisms underlying TRPA1 gene regulation in neuropathic pain. Acrolein is an endogenous aldehyde produced in some nerve lesion models [172,173]. It is known to produce TRPA1-dependent calcium currents in DRG cultures [156], and it upregulated TRPA1 expression in central and peripheral endings of nociceptors [170]. Furthermore, exogenous acrolein, as the one present in tobacco smoke, exacerbated pain in a mouse model of sciatic nerve injury [174]. Oxidative stress produced by nerve lesion may increase acrolein production. Both oxidative stress and acrolein upregulated TRPA1 and could be responsible for the post-traumatic hyperalgesia as pain could be decreased by acrolein scavengers and TRPA1 blockers [172,175]. Regulatory mechanisms of TRPA1 expression in nerve lesion models include p38 MAPK [165,176], ERK, and JNK signalling pathways, [171,177] while PKA and Mass Related G-protein-coupled receptor D upregulate TRPA1 functionality and are essential to establish pain in chronic constriction injury models [178]. Accordingly, in models of neuropathic pain generated by chemotherapeutic agents such as docetaxel, bortezomib, and oxaliplatin, TRPA1 expression is also increased through MAPK signalling [160,179]. Furthermore, TNF-α, released by mast cells, activates p38-MAPK and downstream JNK through TrkA, highlighting the importance of immune system in TRPA1 sensitisation process [180]. In chemotherapy-induced pain models, proteinase activated receptor 2 (PAR2) is another TRPA1 modulator as injection of a PAR2 blocker decreased protein expression of TRPA1 [181], SP, and α-CGRP release [182] and attenuated PKCε and PKA [183]. Additionally, a functional coupling between PAR2 and TRPV1 seems to exist [173]. Finally, in paclitaxel-induced neuropathic pain, TNF-α secreted by glial satellite cells increased TRPA1 and TRPV4 in small size nociceptors [184]. All the mechanisms described above increased TRPA1 expression and consequently α-CGRP and SP secretion, leading to a lower activation threshold of the nociceptors and neurogenic inflammation [185].

### 4.4. Oxidative Stress Is a Major Integrator of TRPA1 Regulation in Neuropathic Pain

Oxidative (ROS) and nitrogen reactive species (RNS) are key factors in the development of neuropathic pain [166,186]. Trevisan et al. described that pain symptoms in a mouse model of nerve injury could be reverted by deleting TRPA1 or blocking TRPA1 receptor. Interestingly, the authors could also reduce pain by injection of an antioxidant (alfa-lipoic acid) or apocynin (a NOX inhibitor), demonstrating that oxidative stress plays a key role in neuropathic pain through TRPA1 action [186]. In accordance, in a saphenous nerve constriction model, TRPA1 and D-amino acid oxidase mRNA were found to be increased in DRGs [166]. Oxidative stress is also necessary in chemotherapy-induced neuropathic pain models. In fact, paclitaxel-induced pain could be abolished by knocking out or blocking both TRPA1 and TRPV4 or by the application of the reducing agent glutathione [164], which also decreased α-CGRP and SP secretion [187]. Furthermore, oxaliplatin-induced cold sensitivity is produced by an increased responsiveness of TRPA1 to ROS. This mechanism is generated by prolyl hydroxylases inhibition [188] whose action on TRPA1 prolynes decreased the channel sensitivity to ROS [34]. Oxaliplatin also acts on cysteine oxidation, which regulated the channel opening confirming that oxidative stress is a pivotal TRPA1 regulator [189]. In the same line, bortezomib-induced neuropathy is characterized by cold, mechanical allodynia and hypersensitivity to a TRPA1 agonist; this effect could be blocked by TRPA1 antagonist HC-03003 or the antioxidant α-lipoic acid [190]. Finally, a recent study found that microRNA 155 mediates TRPA1 upregulation produced by ROS in an oxaliplatin-induced neuropathic pain model [191]. Activation of TRPA1 by oxidative stress has also been seen in other cell types besides primary sensory neurons. In-vivo experiments showed that injection of TRPA1 antagonists or antioxidants in the central nucleus of the amygdala blocked pain produced in the spare nerve injury model in rats [192]. In this regard, TRPA1 expressed in Schwann cells have been described to generate a gradient of oxidative stress, which maintains macrophage-nociceptor communication in mechanical allodynia [193]. In fact, release of H_2_O_2_ and other oxidative stress by-products by macrophage and monocyte has been reported to activate TRPA1 in pain models [167]. Previously mentioned evidence points to ROS-mediated activation of TRPA1 being a major trigger in neuropathic pain; thus antioxidant molecules are being proposed as candidates to treat painful symptoms in neuropathic pain [162,191], such as a blocker of semicarbazide-sensitive amine oxidase (SSAO), TRPA1, and TRPV1 [194].

## 5. TRPA1 in Clinical Trials

To our knowledge, TRPA1 has been implicated in chronic cough [195] and a gain-of-function point mutation has been linked to familial episodic pain syndrome [53]. To date, three TRPA1 antagonists have reached clinical trials for the treatment of pain conditions: GRC1753 (Glenmark) for chronic pain; CB-625 (Cubist Pharmaceuticals Inc.) for acute surgical pain [195,196]; and ODM-108, a highly potent TRPA1 antagonist developed by Orion Pharma to treat neuropathic pain. This drug reached phase 1, but the study failed due to complex pharmacodynamic properties [197]. Finally, acidosis activated human TRPA1 in pathologies such as myocardial infarction or peripheral vascular occlusive disease [198], but it still has to be proven that targeting TRPA1 could alleviate acidosis evoked-pain in clinical trials [199].

## 6. Conclusions

In summary, although TRPV1 has always been the prime TRP channel targeted to develop new drugs to treat pain, accumulating evidences now identify TRPA1 as a potential crucial player in mediating and modulating pain conditions due to its expression in numerous tissues and its key involvement in pivotal signalling pathways. TRPA1 has been demonstrated to cooperate with TRPV1 in the establishment and maintenance of pain and also to be important on its own as a nocisensor of a plethora of molecules. Furthermore, sensitisation of TRPA1 by endogenous mediators and metabolites of oxidative stress position this channel under the spotlight in the study of plenty of diseases, not only in the field of pain. As a consequence, pharmacological inhibition of TRPA1 seems to be an interesting strategy for treating some painful diseases. Further investigation is needed to unveil all the pharmacological potential TRPA1 may have.

## Figures and Tables

**Table 1:** Summary of action sites of the different TRPA1 modulators.

Effect	Region	Residues		Example Substances		References
Electrophilic agonists	Pre-S1 region	C621, C641, C655, K710		Mustard oil, tetrahydrocannabinol, allicin, acrolein, formaldehyde, *N*-methylmaleimide, cinnamaldehyde, eugenol, gingerol, thymol		[41,42,43,44,45,46,47]
Modulators	Ankyrin-repeat domain	C414		Ca^2+^ ions		[48,49,50,51,53]
	S4					
	S1–S4	Negatively charged residues			Tarantula toxin	[52]
Modulator	S5–S6	V875		Menthol		[54]
Nonelectrophilic agonists		S873		Eudesmol		[55]
				Protons		[56]
Antagonists	S5–S6	T874, V876, F877, M956		6-Methyl-5-(2-(trifluoromethyl)phenyl)-1H-indazole		[57]
	Selectivity filter	M911, M912		CMP1, AZ868, HC-030031		[58,59,60,61]
	S6	I940–I950				
	S5	S873, T874	F909	A-967079		[39,62]
				Monoterpens		[63]
Anaesthetics			M912 M953	Propofol, isofluorane		[64]
Modulator	S1–S4	H719, N722, K787, K796, R852, K989		Phosphoinositides		[66]

## References

[1] Eccleston C., Wells C., Morlion B. (2018). European Pain Management.

[2] Gureje O., Simon G.E., Von Korff M. (2001). A cross-national study of the course of persistent pain in primary care. Pain.

[3] Saastamoinen P., Laaksonen M., Kääriä S.M., Lallukka T., Leino-Arjas P., Rahkonen O., Lahelma E. (2012). Pain and disability retirement: a prospective cohort study. Pain.

[4] Dubin A.E., Patapoutian A. (2010). Nociceptors: The sensors of the pain pathway. J. Clin. Investig..

[5] Julius D., Basbaum A.I. (2001). Molecular mechanisms of nociception. Nature.

[6] Belmonte C., Viana F. (2008). Molecular and cellular limits to somatosensory specificity. Mol. Pain.

[7] Woolf C.J. (2004). Pain: Moving from symptom control toward mechanism-specific pharmacologic management. Ann. Intern. Med..

[8] Chaban V.V. (2011). Peripheral sensitization of sensory neurons. Ethn. Dis..

[9] Basbaum A.I., Bautista D.M., Scherrer G., Julius D. (2009). Cellular and Molecular Mechanisms of Pain. Cell.

[10] Ferrer-Montiel A., Fernández-Carvajal A., Planells-Cases R., Fernández-Ballester G., González-Ros J.M., Messeguer A., González-Muñiz R. (2012). Advances in modulating thermosensory TRP channels. Expert Opin. Ther. Pat..

[11] Minke B. (1977). Drosophila mutant with a transducer defect. Biophys. Struct. Mech..

[12] Montell C., Jones K., Hafen E., Rubin G. (1985). Rescue of the Drosophila phototransduction mutation trp by germline transformation. Science.

[13] Levine J.D., Alessandri-Haber N. (2007). TRP channels: targets for the relief of pain. Biochim. Biophys. Acta.

[14] Vandewauw I., De Clercq K., Mulier M., Held K., Pinto S., Van Ranst N., Segal A., Voet T., Vennekens R., Zimmermann K. (2018). A TRP channel trio mediates acute noxious heat sensing. Nature.

[15] Caterina M.J., Schumacher M.A., Tominaga M., Rosen T.A., Levine J.D., Julius D. (1997). The capsaicin receptor: a heat-activated ion channel in the pain pathway. Nature.

[16] Yamamoto Y., Sato Y., Taniguchi K. (2007). Distribution of TRPV1- and TRPV2- immunoreactive afferent nerve endings in rat trachea. J. Anat..

[17] Tatsumi E., Katsura H., Kobayashi K., Yamanaka H., Tsuzuki K., Noguchi K., Sakagami M. (2015). Changes in transient receptor potential channels in the rat geniculate ganglion after chorda tympani nerve injury. Neuroreport.

[18] Jeffry J.A., Yu S.Q., Sikand P., Parihar A., Evans M.S., Premkumar L.S. (2009). Selective targeting of TRPV1 expressing sensory nerve terminals in the spinal cord for long lasting analgesia. PLoS ONE.

[19] Sikand P., Premkumar L.S. (2007). Potentiation of glutamatergic synaptic transmission by protein kinase C-mediated sensitization of TRPV1 at the first sensory synapse. J. Physiol..

[20] Kobayashi K., Fukuoka T., Obata K., Yamanaka H., Dai Y., Tokunaga A., Noguchi K. (2005). Distinct expression of TRPM8, TRPA1, and TRPV1 mRNAs in rat primary afferent neurons with adelta/c fibers and colocalization with trk receptors. J. Comp. Neurol..

[21] Woodbury C.J., Zwick M., Wang S., Lawson J.J., Caterina M.J., Koltzenburg M., Albers K.M., Koerber H.R., Davis B.M. (2004). Nociceptors lacking TRPV1 and TRPV2 have normal heat responses. J. Neurosci..

[22] Davis J.B., Gray J., Gunthorpe M.J., Hatcher J.P., Davey P.T., Overend P., Harries M.H., Latcham J., Clapham C., Atkinson K. (2000). Vanilloid receptor-1 is essential for inflammatory thermal hyperalgesia. Nature.

[23] Caterina M.J., Leffler A., Malmberg A.B., Martin W.J., Trafton J., Petersen-Zeitz K.R., Koltzenburg M., Basbaum A.I., Julius D. (2000). Impaired nociception and pain sensation in mice lacking the capsaicin receptor. Science.

[24] Bolcskei K., Helyes Z., Szabo A., Sandor K., Elekes K., Nemeth J., Almasi R., Pinter E., Petho G., Szolcsanyi J. (2005). Investigation of the role of TRPV1 receptors in acute and chronic nociceptive processes using gene-deficient mice. Pain.

[25] Jaquemar D., Schenker T., Trueb B. (1999). An ankyrin-like protein with transmembrane domains is specifically lost after oncogenic transformation of human fibroblasts. J. Biol. Chem..

[26] Story G.M., Peier A.M., Reeve A.J., Eid S.R., Mosbacher J., Hricik T.R., Earley T.J., Hergarden A.C., Andersson D.A., Hwang S.W. (2003). ANKTM1, a TRP-like channel expressed in nociceptive neurons, is activated by cold temperatures. Cell.

[27] Nagata K., Duggan A., Kumar G., Garcia-Anoveros J. (2005). Nociceptor and hair cell transducer properties of TRPA1, a channel for pain and hearing. J. Neurosci..

[28] Smith M.P., Beacham D., Ensor E., Koltzenburg M. (2004). Cold-sensitive, menthol insensitive neurons in the murine sympathetic nervous system. Neuroreport.

[29] Katsura H., Obata K., Mizushima T., Yamanaka H., Kobayashi K., Dai Y., Fukuoka T., Tokunaga A., Sakagami M., Noguchi K. (2006). Antisense knock down of TRPA1, but not TRPM8, alleviates cold hyperalgesia after spinal nerve ligation in rats. Exp. Neurol..

[30] Zhang X.F., Chen J., Faltynek C.R., Moreland R.B., Neelands T.R. (2008). Transient receptor potential A1 mediates an osmotically activated ion channel. Eur. J. Neurosci..

[31] Bautista D.M., Movahed P., Hinman A., Axelsson H.E., Sterner O., Högestätt E.D., Julius D., Jordt S.E., Zygmunt P.M. (2005). Pungent products from garlic activate the sensory ion channel TRPA. Proc. Natl. Acad. Sci. USA.

[32] Macpherson L.J., Geierstanger B.H., Viswanath V., Bandell M., Eid S.R., Hwang S., Patapoutian A. (2005). The pungency of garlic: activation of TRPA1 and TRPV1 in response to allicin. Curr. Biol..

[33] Babes A., Sauer S.K., Moparthi L., Kichko T.I., Neacsu C., Namer B., Filipovic M., Zygmunt P.M., Reeh P.W., Fischer M.J. (2016). Photosensitization in porphyrias and photodynamic therapy involves TRPA1 and TRPV. J. Neurosci..

[34] Miyake T., Nakamura S., Zhao M., So K., Inoue K., Numata T., Takahashi N., Shirakawa H., Mori Y., Nakagawa T. (2016). Cold sensitivity of TRPA1 is unveiled by the prolyl hydroxylation blockade-induced sensitization to ROS. Nat. Commun..

[35] Bessac B.F., Sivula M., von Hehn C.A., Escalera J., Cohn L., Jordt S.E. (2008). TRPA1 is a major oxidant sensor in murine airway sensory neurons. J. Clin. Investig..

[36] del Camino D., Murphy S., Heiry M., Barrett L.B., Earley T.J., Cook C.A., Petrus M.J., Zhao M., D’Amours M., Deering N. (2010). TRPA1 contributes to cold hypersensitivity. J. Neurosci..

[37] Wang Y.Y., Chang R.B., Waters H.N., McKemy D.D., Liman E.R. (2008). The nociceptor ion cannel TRPA1 is potentiated and inactivated by permeating calcium ions. J. Biol. Chem..

[38] Kwan K.Y., Allchorne A.J., Vollrath M.A., Christensen A.P., Zhang D.S., Woolf C.J., Corey D.P. (2006). TRPA1 contributes to cold, mechanical, and chemical nociception but is not essential for hair-cell transduction. Neuron.

[39] Paulsen C.E., Armache J.P., Gao Y., Cheng Y., Julius D. (2015). Structure of the TRPA1 ion channel suggests regulatory mechanisms. Nature.

[40] Ferrer-Montiel A., García-Martínez C., Morenilla-Palao C., García-Sanz N., Fernández-Carvajal A., Fernández-Ballester G., Planells-Cases R. (2004). Molecular architecture of the vanilloid receptor. Insights for drug design. Eur. J. Biochem..

[41] Macpherson L.J., Dubin A.E., Evans M.J., Marr F., Schultz P.G., Cravatt B.F., Patapoutian A. (2007). Noxious compounds activate TRPA1 ion channels through covalent modification of cysteines. Nature.

[42] Hinman A., Chuang H.H., Bautista D.M., Julius D. (2006). TRP channel activation by reversible covalent modification. Proc. Natl. Acad. Sci. USA.

[43] Jordt S.E., Bautista D.M., Chuang H.H., McKemy D.D., Zygmunt P.M., Högestätt E.D., Meng I.D., Julius D. (2004). Mustard oils and cannabinoids excite sensory nerve fibres through the TRP channel ANKTM. Nature.

[44] Bautista D.M., Jordt S.E., Nikai T., Tsuruda P.R., Read A.J., Poblete J., Yamoah E.N., Basbaum A.I., Julius D. (2006). TRPA1 mediates the inflammatory actions of environmental irritants and proalgesic agents. Cell.

[45] Bandell M., Story G.M., Hwang S.W., Viswanath V., Eid S.R., Petrus M.J., Earley T.J., Patapoutian A. (2004). Noxious cold ion channel TRPA1 is activated by pungent compounds and bradykinin. Neuron.

[46] Mendes S.J.F., Sousa F.I.A.B., Pereira D.M.S., Ferro T.A.F., Pereira I.C.P., Silva B.L.R., Pinheiro A.J.M.C.R., Mouchrek A.Q.S., Monteiro-Neto V., Costa S.K.P. (2016). Cinnamaldehyde modulates LPS-induced systemic inflammatory response syndrome through TRPA1-dependent and independent mechanisms. Int. Immunopharmacol..

[47] Xu H., Delling M., Jun J.C., Clapham D.E. (2006). Oregano, thyme and clove-derived flavors and skin sensitizers activate specific TRP channels. Nat. Neurosci..

[48] Cavanaugh E.J., Simkin D., Kim D. (2008). Activation of transient receptor potential A1 channels by mustard oil, tetrahydrocannabinol and Ca^2+^ reveals different functional channel states. Neuroscience.

[49] Zurborg S., Yurgionas B., Jira J.A., Caspani O., Heppenstall P.A. (2007). Direct activation of the ion channel TRPA1 by Ca^2+^. Nat. Neurosci..

[50] Takahashi N., Mizuno Y., Kozai D., Yamamoto S., Kiyonaka S., Shibata T., Uchida K., Mori Y. (2008). Molecular characterization of TRPA1 channel activation by cysteine-reactive inflammatory mediators. Channels.

[51] Marsakova L., Barvik I., Zima V., Zimova L., Vlachova V. (2017). The First Extracellular Linker Is Important for Several Aspects of the Gating Mechanism of Human TRPA1 Channel. Front. Mol. Neurosci..

[52] Gui J., Liu B., Cao G., Lipchik A.M., Perez M., Dekan Z., Mobli M., Daly N.L., Alewood P.F., Parker L.L. (2014). A tarantula-venom peptide antagonizes the TRPA1 nociceptor ion channel by binding to the S1–S4 gating domain. Curr. Biol..

[53] Kremeyer B., Lopera F., Cox J.J., Momin A., Rugiero F., Marsh S., Woods C.G., Jones N.G., Paterson K.J., Fricker F.R. (2010). A gain-of-function mutation in TRPA1 causes familial episodic pain syndrome. Neuron.

[54] Xiao B., Dubin A.E., Bursulaya B., Viswanath V., Jegla T.J., Patapoutian A. (2008). Identification of transmembrane domain 5 as a critical molecular determinant of menthol sensitivity in mammalian TRPA1 channels. J. Neurosci..

[55] Ohara K., Fukuda T., Okada H., Kitao S., Ishida Y., Kato K., Takahashi C., Katayama M., Uchida K., Tominaga M. (2015). Identification of significant amino acids in multiple transmembrane domains of human transient receptor potential ankyrin 1 (TRPA1) for activation by eudesmol, an oxygenized sesquiterpene in hop essential oil. J. Biol. Chem..

[56] de la Roche J., Eberhardt M.J., Klinger A.B., Stanslowsky N., Wegner F., Koppert W., Reeh P.W., Lampert A., Fischer M.J., Leffler A. (2013). The molecular basis for species-specific activation of human TRPA1 protein by protons involves poorly conserved residues within transmembrane domains 5 and 6. J. Biol. Chem..

[57] Moldenhauer H., Latorre R., Grandl J. (2014). The pore-domain of TRPA1 mediates the inhibitory effect of the antagonist 6-methyl-5-(2-(trifluoromethyl)phenyl)-1H-indazole. PLoS ONE.

[58] Chen J., Zhang X.F., Kort M.E., Huth J.R., Sun C., Miesbauer L.J., Cassar S.C., Neelands T., Scott V.E., Moreland R.B. (2008). Molecular determinants of species-specific activation or blockade of TRPA1 channels. J. Neurosci..

[59] Vallin K.S., Sterky K.J., Nyman E., Bernström J., From R., Linde C., Minidis A.B., Nolting A., Närhi K., Santangelo E.M. (2012). N-1-Alkyl-2-oxo-2-aryl amides as novel antagonists of the TRPA1 receptor. Bioorg. Med. Chem. Lett..

[60] Eid S.R., Crown E.D., Moore E.L., Liang H.A., Choong K.C., Dima S., Henze D.A., Kane S.A., Urban M. (2008). O HC-030031, a TRPA1 selective antagonist, attenuates inflammatory- and neuropathy-induced mechanical hypersensitivity. Mol. Pain.

[61] Klement G., Eisele L., Malinowsky D., Nolting A., Svensson M., Terp G., Weigelt D., Dabrowski M. (2013). Characterization of a ligand binding site in the human transient receptor potential ankyrin 1 pore. Biophys. J..

[62] Chen J., Joshi S.K., DiDomenico S., Perner R.J., Mikusa J.P., Gauvin D.M., Segreti J.A., Han P., Zhang X.F., Niforatos W. (2011). Selective blockade of TRPA1 channel attenuates pathological pain without altering noxious cold sensation or body temperature regulation. Pain.

[63] Takaishi M., Uchida K., Fujita F., Tominaga M. (2014). Inhibitory effects of monoterpenes on human TRPA1 and the structural basis of their activity. J. Physiol. Sci..

[64] Ton H.T., Phan T.X., Abramyan A.M., Shi L., Ahern G.P. (2017). Identification of a putative binding site critical for general anesthetic activation of TRPA. Proc. Natl. Acad. Sci. USA.

[65] Christensen A.P., Akyuz N., Corey D.P. (2016). The Outer Pore and Selectivity Filter of TRPA. PLoS ONE.

[66] Zimova L., Sinica V., Kadkova A., Vyklicka L., Zima V., Barvik I., Vlachova V. (2018). Intracellular cavity of sensor domain controls allosteric gating of TRPA1 channel. Sci. Signal..

[67] Banke T.G., Chaplan S.R., Wickenden A.D. (2010). Dynamic changes in the TRPA1 selectivity filter lead to progressive but reversible pore dilation. Am. J. Physiol. Cell Physiol..

[68] Chen J., Kim D., Bianchi B.R., Cavanaugh E.J., Faltynek C.R., Kym P.R., Reilly R.M. (2009). Pore dilation occurs in TRPA1 but not in TRPM8 channels. Mol. Pain.

[69] Ferreira L.G., Faria R.X. (2016). TRPing on the pore phenomenon: what do we know about transient receptor potential ion channel-related pore dilation up to now?. J. Bioenerg. Biomembr..

[70] Biggs J.E., Stemkowski P.L., Knaus E.E., Chowdhury M.A., Ballanyi K., Smith P.A. (2015). Suppression of network activity in dorsal horn by gabapentin permeation of TRPV1 channels: implications for drug access to cytoplasmic targets. Neurosci. Lett..

[71] Banke T.G. (2011). The dilated TRPA1 channel pore state is blocked by amiloride and analogues. Brain Res..

[72] Sousa-Valente J., Andreou A.P., Urban L., Nagy I. (2014). Transient receptor potential ion channels in primary sensory neurons as targets for novel analgesics. Br. J. Pharmacol..

[73] Patapoutian A., Tate S., Woolf C.J. (2009). Transient receptor potential channels: targeting pain at the source. Nat. Rev. Drug. Discov..

[74] Vay L., Gu C., McNaughton P.A. (2012). The thermos-TRP ion channel family: properties and therapeutic implications. Br. J. Pharmacol..

[75] Holtzer P. (2011). Transient receptor potential (TRP) channels as drug targets for diseases of the digestive system. Pharmacol. Ther..

[76] Schmidt M., Dubin A.E., Petrus M.J., Earley T.J., Patapoutian A. (2009). Nociceptive signals induce trafficking of TRPA1 to the plasma membrane. Neuron.

[77] Brierly S.M., Hughes P.A., Page A.J., Kwan K.Y., Martin C.M., O’Donnell T.A., Cooper N.J., Harrington A.M., Adam B., Liebregts T. (2009). The Ion Channel TRPA1 Is Required for Normal Mechanosensation and Is Modulated by Algesic Stimuli. Gastroenterology.

[78] Nozawa K., Kawabata-shoda E., Doihara H., Kojima R., Okada H., Mochizuki S., Sano Y., Inamura K., Matsushime H., Koizumi T. (2009). TRPA1 regulates gastrointestinal motility through serotonin release from enterochromaffin cells. Proc. Natl. Acad. Sci. USA.

[79] Zielińska M., Jarmuz A., Wasilewski A., Sałaga M., Fichna J. (2015). Role of transient receptor potential channels in intestinal inflammation and visceral pain: Novel targets in inflammatory bowel diseases. Inflamm. Bowel. Dis..

[80] Feng C., Yan X., Chen X., Wang E., Liu Q., Zhang L., Chen J., Fang J.Y., Chen S. (2014). Vagal anandamide signaling via cannabinoid receptor 1 contributes to luminal 5-HT modulation of visceral nociception in rats. Pain.

[81] Malin S., Molliver D., Christianson J.A., Schwartz E.S., Cornuet P., Albers K.M., Davis B.M. (2011). TRPV1 and TRPA1 Function and Modulation are Target Tissue- Dependent. J. Neurosci..

[82] Shen S., Al-thumairy H.W., Hashmi F., Qiao L. (2017). Regulation of transient receptor potential cation channel subfamily V1 protein synthesis by the phosphoinositide 3- kinase/Akt pathway in colonic hypersensitivity. Exp. Neurol..

[83] Kogure Y., Wang S., Tanaka K.I., Hao Y., Yamamoto S., Nishiyama N., Noguchi K., Dai Y. (2016). Elevated H_2_O_2_ levels in trinitrobenzene sulfate-induced colitis rats contributes to visceral hyperalgesia through interaction with the transient receptor potential ankyrin 1 cation channel. J. Gastroenterol. Hepatol..

[84] Yang J., Li Y., Zuo X., Zhen Y., Yu Y., Gao L. (2008). Transient receptor potential ankyrin-1 participates in visceral hyperalgesia following experimental colitis. Neurosci. Lett..

[85] Kistner K., Siklosi N., Babes A., Khalil M., Selescu T., Zimmermann K., Wirtz S., Becker C., Neurath M.F., Reeh P.W. (2016). Systemic desensitization through TRPA1 channels by capsazepine and mustard oil—A novel strategy against inflammation and pain. Sci. Rep..

[86] Engel M.A., Leffler A., Niedermirtl F., Babes A., Tribbensee S.M.M., Khalil M., Siklosi N., Nau C., Ivanović-Burmazović I., Neuhuber W.L. (2011). TRPA1 and Substance P Mediate Colitis in Mice. Gastroenterology.

[87] Romano B., Borrelli F., Fasolino I., Capasso R., Piscitelli F., Ii F., Domenico V. (2013). The cannabinoid TRPA1 agonist cannabichromene inhibits nitric oxide production in macrophages and ameliorates murine colitis. Br. J. Pharmacol..

[88] Kojima R., Nozawa K., Doihara H., Keto Y., Kaku H. (2014). Effects of novel TRPA1 receptor agonist ASP7663 in models of drug-induced constipation and visceral pain. Eur. J. Pharmacol..

[89] Mitrovic M., Shahbazian A., Bock E., Pabst M.A., Holzer P. (2010). Chemo-nociceptive signalling from the colon is enhanced by mild colitis and blocked by inhibition of transient receptor potential ankyrin 1 channels. Br. J. Pharmacol..

[90] Alvarenga E.M., Souza L.K.M., Araújo T.S.L., Nogueira K.M., Sousa F.B.M., Araújo A.R., Martins C., Pacífico D.M., de C Brito G.A., Souza E.P. (2016). Carvacrol reduces irinotecan-induced intestinal mucositis through inhibition of inflammation and oxidative damage via TRPA1 receptor activation. Chem. Biol. Interact..

[91] Kurahara L.H., Hiraishi K., Hu Y., Koga K., Onitsuka M., Doi M., Aoyagi K., Takedatsu H., Kojima D., Fujihara Y. (2018). Activation of Myofibroblast TRPA1 by Steroids and Pirfenidone Ameliorates Fibrosis in Experimental Crohn’s Disease. Cell. Mol. Gastroenterol. Hepatol..

[92] Koivisto A., Jalava N., Bratty R., Pertovaara A. (2018). TRPA1 antagonists for pain relief. Pharmaceuticals.

[93] Nummenmaa E., Hämäläinen M., Moilanen L.J., Paukkeri E.L., Nieminen R.M., Moilanen T., Vuolteenaho K., Moilanen E. (2016). Transient receptor potential ankyrin 1 (TRPA1) is functionally expressed in primary human osteoarthritic chondrocytes. Arthritis Res. Ther..

[94] Horváth Á., Tékus V., Boros M., Pozsgai G., Botz B., Borbély É., Szolcsányi J., Pintér E., Helyes Z. (2016). Transient receptor potential ankyrin 1 (TRPA1) receptor is involved in chronic arthritis: In vivo study using TRPA1-deficient mice. Arthritis Res. Ther..

[95] Moilanen L.J., Hämäläinen M., Nummenmaa E., Ilmarinen P., Vuolteenaho K., Nieminen R.M., Lehtimäki L., Moilanen E. (2015). Monosodium iodoacetate-induced inflammation and joint pain are reduced in TRPA1 deficient mice - potential role of TRPA1 in osteoarthritis. Osteoarthr. Cartil..

[96] Fernandes E.S., Russell F.A., Spina D., McDougall J.J., Graepel R., Gentry C., Staniland A.A., Mountford D.M., Keeble J.E., Malcangio M. (2011). A distinct role for transient receptor potential ankyrin 1, in addition to transient receptor potential vanilloid 1, in tumor necrosis factorα-induced inflammatory hyperalgesia and Freund’s complete adjuvant-induced monarthritis. Arthritis Rheum..

[97] Pereira L.M.S., Lima-Júnior R.C.P., Bem A.X.C., Teixeira C.G., Grassi L.S., Medeiros R.P., Marques-Neto R.D., Callado R.B., Aragão K.S., Wong D.V. (2013). Blockade of TRPA1 with HC-030031 attenuates visceral nociception by a mechanism independent of inflammatory resident cells, nitric oxide and the opioid system. Eur. J. Pain.

[98] Lowin T., Bleck J., Schneider M., Pongratz G. (2018). Selective killing of proinflammatory synovial fibroblasts via activation of transient receptor potential ankyrin (TRPA1). Biochem. Pharmacol..

[99] Lowin T., Apitz M., Anders S., Straub R.H. (2015). Anti-inflammatory effects of N-acylethanolamines in rheumatoid arthritis synovial cells are mediated by TRPV1 and TRPA1 in a COX-2 dependent manner. Arthritis Res. Ther..

[100] Dalbeth N., Merriman T.R., Stamp L.K. (2016). Gout. Lancet.

[101] Moilanen L.J., Hämäläinen M., Lehtimäki L., Nieminen R.M., Moilanen E. (2015). Urate crystal induced inflammation and joint pain are reduced in transient receptor potential ankyrin 1 deficient mice - Potential role for transient receptor potential ankyrin1 in gout. PLoS ONE.

[102] Maruyama K., Takayama Y., Kondo T., Ishibashi K., Sahoo B.R., Kanemaru H., Kumagai Y., Martino M.M., Tanaka H., Ohno N. (2017). Nociceptors Boost the Resolution of Fungal Osteoinflammation via the TRP Channel-CGRP-Jdp2 Axis. Cell Rep..

[103] Asgar J., Zhang Y., Saloman J.L., Wang S., Chung M.K., Ro J.Y. (2015). The role of TRPA1 in muscle pain and mechanical hypersensitivity under inflammatory conditions in rats. Neuroscience.

[104] Chung M.K., Park J., Asgar J., Ro J.Y. (2016). Transcriptome analysis of trigeminal ganglia following masseter muscle inflammation in rats. Mol. Pain.

[105] Diogenes A., Akopian A.N., Hargreaves K.M. (2007). NGF Up-regulates TRPA1: Implications for orofacial pain. J. Dent. Res..

[106] Wang S., Brigoli B., Lim J., Karley A., Chung M.K. (2018). Roles of TRPV1 and TRPA1 in Spontaneous Pain from Inflamed Masseter Muscle. Neuroscience.

[107] Sugiyama D., Kang S., Brennan T.J. (2017). Muscle reactive oxygen species (ROS) contribute to post-incisional guarding via the TRPA1 receptor. PLoS ONE.

[108] Iannotti F.A., Pagano E., Moriello A.S., Alvino F.G., Sorrentino N.C., D’Orsi L., Gazzerro E., Capasso R., De Leonibus E., De Petrocellis L. (2019). Effects of non-euphoric plant cannabinoids on muscle quality and performance of dystrophic mdx mice. Br. J. Pharmacol..

[109] Wilson S.R., Nelson A.M., Batia L., Morita T., Estandian D., Owens D.M., Lumpkin E.A., Bautista D.M. (2013). The Ion Channel TRPA1 Is Required for Chronic Itch. J. Neurosci..

[110] Kodji X., Arkless K.L., Kee Z., Cleary S.J., Aubdool A.A., Evans E., Caton P., Pitchford S.C., Brain S.D. (2019). Sensory nerves mediate spontaneous behaviors in addition to inflammation in a murine model of psoriasis. FASEB J..

[111] Fernandes E.S., Vong C.T., Quek S., Cheong J., Awal S., Gentry C., Aubdool A.A., Liang L., Bodkin J.V., Bevan S. (2013). Superoxide generation and leukocyte accumulation: Key elements in the mediation of leukotriene B4-induced itch by transient receptor potential ankyrin 1 and transient receptor potential vanilloid 1. FASEB J..

[112] Cevikbas F., Wang X., Akiyama T., Kempkes C., Savinko T., Antal A., Kukova G., Buhl T., Ikoma A., Buddenkotte J. (2014). sensory neuron-expressed IL-31 receptor mediates T helper cell-dependent itch: Involvement of TRPV1 and TRPA. J. Allergy Clin. Immunol..

[113] Liu B., Escalera J., Balakrishna S., Fan L., Caceres A.I., Robinson E., Sui A., McKay M.C., McAlexander M.A., Herrick C.A. (2013). TRPA1 controls inflammation and pruritogen responses in allergic contact dermatitis. FASEB J..

[114] Liu T., Ru-Rong J. (2012). Oxidative stress induces itch via activation of transient receptor potential subtype ankyrin 1 in mice. Neurosci. Bull..

[115] Wilson S.R., Gerhold K.A., Bifolck-Fisher A., Liu Q., Patel K.N., Dong X., Bautista D.M. (2011). TRPA1 is required for histamine-independent, Mas-related G protein-coupled receptor-mediated itch. Nat. Neurosci..

[116] Feng J., Yang P., Mack M.R., Dryn D., Luo J., Gong X., Liu S., Oetjen L.K., Zholos A.V., Mei Z. (2017). Sensory TRP channels contribute differentially to skin inflammation and persistent itch. Nat. Commun..

[117] Oh M.H., Oh S.Y., Lu J., Lou H., Myers A.C., Zhu Z., Zheng T. (2013). TRPA1-Dependent Pruritus in IL-13-Induced Chronic Atopic Dermatitis. J. Immunol..

[118] Moore C., Gupta R., Jordt S.E., Chen Y., Liedtke W.B. (2018). Regulation of Pain and Itch by TRP Channels. Neurosci. Bull..

[119] Conklin D.J. (2016). Acute cardiopulmonary toxicity of inhaled aldehydes: role of TRPA. Ann. N. Y. Acad. Sci..

[120] Page C.P., Barnes P.J. (2017). Pharmacology and Therapeutics of Asthma and COPD. Handbook of Experimental Pharmacology.

[121] Zholos A., McGarvey L., Ennis M. (2015). TRP Channels as Therapeutic Targets: From Basic Science to Clinical Use.

[122] Taylor-Clark T.E., Undem B.J. (2010). Ozone activates airway nerves via the selective stimulation of TRPA1 ion channels. J. Physiol..

[123] Lin A.H., Liu M.H., Ko H.K., Perng D.W., Lee T.S., Kou Y.R. (2015). Lung Epithelial TRPA1 Transduces the Extracellular ROS into Transcriptional Regulation of Lung Inflammation Induced by Cigarette Smoke: The Role of Influxed Ca^2+^. Mediat. Inflamm..

[124] Kortekaas Krohn I., Callebaut I., Alpizar Y.A., Steelant B., Van Gerven L., Skov P.S., Kasran A., Talavera K., Wouters M.M., Ceuppens J.L. (2018). MP29-02 reduces nasal hyperreactivity and nasal mediators in patients with house dust mite-allergic rhinitis. Allergy.

[125] Hsu C.C., Lee L.Y. (2015). Role of calcium ions in the positive interaction between TRPA1 and TRPV1 channels in bronchopulmonary sensory neurons. J. Appl. Physiol..

[126] Lin Y.J., Lin R.L., Ruan T., Khosravi M., Lee L.Y. (2015). A synergistic effect of simultaneous TRPA1 and TRPV1 activations on vagal pulmonary C-fiber afferents. J. Appl. Physiol..

[127] Alenmyr L., Herrmann A., Högestätt E.D., Greiff L., Zygmunt P.M. (2011). TRPV1 and TRPA1 stimulation induces MUC5B secretion in the human nasal airway in vivo. Clin. Physiol. Funct. Imaging.

[128] Prandini P., De Logu F., Fusi C., Provezza L., Nassini R., Montagner G., Materazzi S., Munari S., Gilioli E., Bezzerri V. (2016). Transient receptor potential ankyrin 1 channels modulate inflammatory response in respiratory cells from patients with cystic fibrosis. Am. J. Respir. Cell Mol. Biol..

[129] Streng T., Axelsson H.E., Hedlund P., Andersson D.A., Jordt S.E., Bevan S., Andersson K.E., Högestätt E.D., Zygmunt P.M. (2008). Distribution and Function of the Hydrogen Sulfide-Sensitive TRPA1 Ion Channel in Rat Urinary Bladder. Eur. Urol..

[130] Silva R.B.M., Sperotto N.D.M., Andrade E.L., Pereira T.C.B., Leite C.E., De Souza A.H., Bogo M.R., Morrone F.B., Gomez M.V., Campos M.M. (2015). Spinal blockage of P/Q-or N-type voltage-gated calcium channels modulates functional and symptomatic changes related to haemorrhagic cystitis in mice. Br. J. Pharmacol..

[131] Chen Z., Du S., Kong C., Zhang Z., Mokhtar A.D. (2016). Intrathecal administration of TRPA1 antagonists attenuate cyclophosphamide-induced cystitis in rats with hyper-reflexia micturition. BMC Urol..

[132] Meotti F.C., Forner S., Lima-Garcia J.F., Viana A.F., Calixto J.B. (2013). Antagonism of the transient receptor potential ankyrin 1 (TRPA1) attenuates hyperalgesia and urinary bladder overactivity in cyclophosphamide-induced haemorrhagic cystitis. Chem. Biol. Interact..

[133] Andrade E.L., Forner S., Bento A.F., Ferraz D., Leite P., Dias M.A., Leal P.C., Koepp J., Calixto J.B. (2011). TRPA1 receptor modulation attenuates bladder overactivity induced by spinal cord injury. Am. J. Physiol. Renal. Physiol..

[134] Kamei J., Aizawa N., Nakagawa T., Kaneko S., Kume H., Homma Y., Igawa Y. (2018). Attenuated lipopolysaccharide-induced inflammatory bladder hypersensitivity in mice deficient of transient receptor potential ankilin. Sci. Rep..

[135] Oyama S., Dogishi K., Kodera M., Kakae M., Nagayasu K., Shirakawa H., Nakagawa T., Kaneko S. (2017). Pathophysiological role of transient receptor potential ankyrin 1 in a mouse long-lasting cystitis model induced by an intravesical injection of hydrogen peroxide. Front. Physiol..

[136] Chanpimol S., Seamon B., Hernandez H., Harris-love M., Blackman M.R. (2017). Effects of Estrogen Receptor β Stimulation in a Rat Model of Non-Bacterial Prostatic Inflammation. Prostate.

[137] Lim J.Y., Park C.K., Hwang S.W. (2015). Biological Roles of Resolvins and Related Substances in the Resolution of Pain. Biomed. Res. Int..

[138] McNamara C.R., Mandel-Brehm J., Bautista D.M., Siemens J., Deranian K.L., Zhao M., Hayward N.J., Chong J.A., Julius D., Moran M.M. (2007). TRPA1 mediates formalin-induced pain. Proc. Natl. Acad. Sci. USA.

[139] Yue Z., Xie J., Yu A., Stock J., Du J., Yue L. (2014). Role of TRP channels in the cardiovascular system. Am. J. Physiol. Heart Circ. Physiol..

[140] Zhao J.F., Shyue S.K., Kou Y.R., Lu T.M., Lee T.S. (2016). Transient receptor potential ankyrin 1 channel involved in atherosclerosis and macrophage-foam cell formation. Int. J. Biol. Sci..

[141] Ogawa N., Kurokawa T., Mori Y. (2016). Sensing of redox status by TRP channels. Cell Calcium.

[142] Oehler B., Kistner K., Martin C., Schiller J., Mayer R., Mohammadi M., Sauer R.S., Filipovic M.R., Nieto F.R., Kloka J. (2017). Inflammatory pain control by blocking oxidized phospholipid-mediated TRP channel activation. Sci. Rep..

[143] Wang Z., Wang M., Liu J., Ye J., Jiang H., Xu Y., Ye D., Wan J. (2018). Inhibition of TRPA1 attenuates doxorubicin-induced acute cardiotoxicity by suppressing oxidative stress, the inflammatory response, and endoplasmic reticulum stress. Oxid. Med. Cell. Longev..

[144] Wang Z., Xu Y., Wang M., Ye J., Liu J., Jiang H., Ye D., Wan J. (2018). TRPA1 inhibition ameliorates pressure overload-induced cardiac hypertrophy and fibrosis in mice. EBioMedicine.

[145] Mergler S., Valtink M., Takayoshi S., Okada Y., Miyajima M., Saika S., Reinach P.S. (2014). Temperature-sensitive transient receptor potential channels in corneal tissue layers and cells. Ophthalmic. Res..

[146] Acosta C.M., Luna C., Quirce S., Belmonte C., Gallar J. (2014). Corneal sensory nerve activity in an experimental model of UV keratitis. Investig. Ophthalmol. Vis. Sci..

[147] Okada Y., Shirai K., Reinach P.S., Kitano-Izutani A., Miyajima M., Flanders K.C., Jester J.V., Tominaga M., Saika S. (2014). TRPA1 is required for TGF-β signaling and its loss blocks inflammatory fibrosis in mouse corneal stroma. Lab. Investig..

[148] Okada Y., Reinach P., Shirai K., Kitano-Izutani A., Miyajima M., Yamanaka O., Sumioka T., Saika S. (2015). Transient Receptor Potential Channels and Corneal Stromal Inflammation. Cornea.

[149] Katagiri A., Thompson R., Rahman M., Okamoto K., Bereiter D.A. (2015). Evidence for TRPA1 involvement in central neural mechanisms in a rat model of dry eye. Neuroscience.

[150] Jensen T.S., Madsen C.S., Finnerup N.B. (2009). Pharmacology and treatment of neuropathic pains. Curr. Opin. Neurol..

[151] Brix Finnerup N., Hein Sindrup S., Staehelin Jensen T. (2013). Management of painful neuropathies. Handb. Clin. Neurol..

[152] Andrade E.L., Meotti F.C., Calixto J.B. (2012). TRPA1 antagonists as potential analgesic drugs. Pharmacol. Ther..

[153] Story G.M., Gereau R.W. (2006). Numbing the senses: role of TRPA1 in mechanical and cold sensation. Neuron.

[154] Demartini C., Greco R., Zanaboni A.M., Francesconi O., Nativi C., Tassorelli C., Deseure K. (2018). Antagonism of Transient Receptor Potential Ankyrin Type-1 Channels as a Potential Target for the Treatment of Trigeminal Neuropathic Pain: Study in an Animal Model. Int. J. Mol. Sci..

[155] Damasceno M.B., de Melo Júnior J.M., Santos S.A., Melo L.T., Leite L.H., Vieira-Neto A.E., Moreira R.A., Monteiro-Moreira A.C., Campos A.R. (2016). Frutalin reduces acute and neuropathic nociceptive behaviours in rodent models of orofacial pain. Chem. Biol. Interact..

[156] Nassini R., Fusi C., Materazzi S., Coppi E., Tuccinardi T., Marone I.M., De Logu F., Preti D., Tonello R., Chiarugi A. (2015). The TRPA1 channel mediates the analgesic action of dipyrone and pyrazolone derivatives. Br. J. Pharmacol..

[157] Kim S.H., Kim W., Kim J.H., Woo M.K., Baek J.Y., Kim S.Y., Chung S.H., Kim H.J. (2018). A Prospective Study of Chronic Oxaliplatin-Induced Neuropathy in Patients with Colon Cancer: Long-Term Outcomes and Predictors of Severe Oxaliplatin-Induced Neuropathy. J. Clin. Neurol..

[158] Authier N., Balayssac D., Marchand F., Ling B., Zangarelli A., Descoeur J., Coudore F., Bourinet E., Eschalier A. (2009). Animal models of chemotherapy-evoked painful peripheral neuropathies. Neurotherapeutics.

[159] Zhou H.H., Zhang L., Zhou Q.G., Fang Y., Ge W.H. (2016). (+)-Borneol attenuates oxaliplatin-induced neuropathic hyperalgesia in mice. NeuroReport.

[160] Huang K., Bian D., Jiang B., Zhai Q., Gao N., Wang R. (2017). TRPA1 contributed to the neuropathic pain induced by docetaxel treatment. Cell. Biochem. Funct..

[161] Zhao M., Isami K., Nakamura S., Shirakawa H., Nakagawa T., Kaneko S. (2012). Acute cold hypersensitivity characteristically induced by oxaliplatin is caused by the enhanced responsiveness of TRPA1 in mice. Mol. Pain.

[162] Nassini R., Gees M., Harrison S., De Siena G., Materazzi S., Moretto N., Failli P., Preti D., Marchetti N., Cavazzini A. (2011). Oxaliplatin elicits mechanical and cold allodynia in rodents via TRPA1 receptor stimulation. Pain.

[163] Park J.H., Chae J., Roh K., Kil E.J., Lee M., Auh C.K., Lee M.A., Yeom C.H., Lee S. (2015). Oxaliplatin-induced Peripheral Neuropathy via TRP1 Stimulation in Mice Dorsal Root Ganglion Is Correlated with Aluminium Accumulation. PLoS ONE.

[164] Lee M., Cho S., Roh K., Chae J., Park J.H., Park J., Lee M.A., Kim J., Auh C.K., Yeom C.H. (2017). Glutathione alleviated peripheral neuropathy in oxaliplatin-treated mice by removing aluminum from dorsal root ganglia. Am. J. Transl. Res..

[165] Obata K., Yamanaka H., Dai Y., Mizushima T., Fukuoka T., Tokunaga A., Noguchi K. (2004). Differential activation of MAPK in injured and uninjured DRG neurons following chronic constriction injury of the sciatic nerve in rats. Eur. J. Neurosci..

[166] Wei H., Wu H.Y., Chen Z., Ma A.N., Mao X.F., Li T.F., Li X.Y., Wang Y.X., Pertovaara A. (2016). Mechanical antihypersensitivity effect induced by repeated spinal administrations of a TRPA1 antagonist or a gap junction decoupler in peripheral neuropathy. Pharmacol. Biochem. Behav..

[167] Chukyo A., Chiba T., Kambe T., Yamamoto K., Kawakami K., Taguchi K., Abe K. (2018). Oxaliplatin-induced changes in expression of transient receptor potential channels in the dorsal root ganglion as a neuropathic mechanism for cold hypersensitivity. Neuropeptides.

[168] Xie H.T., Xia Z.Y., Pan X., Zhao B., Liu Z.G. (2018). Puerarin ameliorates allodynia and hyperalgesia in rats with peripheral nerve injury. Neural. Regen. Res..

[169] Lu S., Ma S., Wang Y., Huang T., Zhu Z., Zhao G. (2017). Mus musculus-microRNA-449a ameliorates neuropathic pain by decreasing the level of KCNMA1 and TRPA1, and increasing the level of TPTE. Mol. Med. Rep..

[170] Park J., Zheng L., Acosta G., Vega-Alvarez S., Chen Z., Muratori B., Cao P., Shi R. (2015). Acrolein contributes to TRPA1 up-regulation in peripheral and central sensory hypersensitivity following spinal cord injury. J. Neurochem..

[171] Zhuang Z.Y., Wen Y.R., Zhang D.R., Borsello T., Bonny C., Strichartz G.R., Decosterd I., Ji R.R. (2006). A peptide c-Jun N-terminal kinase (JNK) inhibitor blocks mechanical allodynia after spinal nerve ligation: respective roles of JNK activation in primary sensory neurons and spinal astrocytes for neuropathic pain development and maintenance. J. Neurosci..

[172] Lin Y., Chen Z., Tang J., Cao P., Shi R. (2017). Acrolein Contributes to the Neuropathic Pain and Neuron Damage after Ischemic-Reperfusion Spinal Cord Injury. Neuroscience.

[173] Chen Y., Yang C., Wang Z.J. (2011). Proteinase-activated receptor 2 sensitizes transient receptor potential vanilloid 1, transient receptor potential vanilloid 4, and transient receptor potential ankyrin 1 in paclitaxel-induced neuropathic pain. Neuroscience.

[174] Butler B., Acosta G., Shi R. (2017). Exogenous Acrolein intensifies sensory hypersensitivity after spinal cord injury in rat. J. Neurol. Sci..

[175] Chen Z., Park J., Butler B., Acosta G., Vega-Alvarez S., Zheng L., Tang J., McCain R., Zhang W., Ouyang Z. (2016). Mitigation of sensory and motor deficits by acrolein scavenger phenelzine in a rat model of spinal cord contusive injury. J. Neurochem..

[176] Schäfers M., Svensson C.I., Sommer C., Sorkin L.S. (2003). Tumor necrosis factor-alpha induces mechanical allodynia after spinal nerve ligation by activation of p38 MAPK in primary sensory neurons. J. Neurosci..

[177] Obata K., Yamanaka H., Kobayashi K., Dai Y., Mizushima T., Katsura H., Fukuoka T., Tokunaga A., Noguchi K. (2004). Role of mitogen-activated protein kinase activation in injured and intact primary afferent neurons for mechanical and heat hypersensitivity after spinal nerve ligation. J. Neurosci..

[178] Wang C., Gu L., Ruan Y., Geng X., Xu M., Yang N., Yu L., Jiang Y., Zhu C., Yang Y. (2019). Facilitation of MrgprD by TRP-A1 promotes neuropathic pain. FASEB J..

[179] Yamamoto K., Chiba N., Chiba T., Kambe T., Abe K., Kawakami K., Utsunomiya I., Taguchi K. (2015). Transient receptor potential ankyrin 1 that is induced in dorsal root ganglion neurons contributes to acute cold hypersensitivity after oxaliplatin administration. Mol. Pain.

[180] Li C., Deng T., Shang Z., Wang D., Xiao Y. (2018). Blocking TRPA1 and TNF-α Signal Improves Bortezomib-Induced Neuropathic Pain. Cell Physiol. Biochem..

[181] Tian L., Fan T., Zhou N., Guo H., Zhang W. (2015). Role of PAR2 in regulating oxaliplatin-induced neuropathic pain via TRPA. Transl. Neurosci..

[182] Chen K., Zhang Z.F., Liao M.F., Yao W.L., Wang J., Wang X.R. (2015). Blocking PAR2 attenuates oxaliplatin-induced neuropathic pain via TRPV1 and releases of substance P and CGRP in superficial dorsal horn of spinal cord. J. Neurol. Sci..

[183] Wang Q., Wang Gao D., Li J. (2017). Inhibition of PAR2 and TRPA1 signals alleviates neuropathic pain evoked by chemotherapeutic bortezomib. J. Biol. Regul. Homeost. Agents.

[184] Wu Z., Wang S., Wu I., Mata M., Fink D.J. (2015). Activation of TLR-4 to produce tumour necrosis factor-α in neuropathic pain caused by paclitaxel. Eur. J. Pain.

[185] Nassini R., Materazzi S., Benemei S., Geppetti P. (2014). The TRPA1 channel in inflammatory and neuropathic pain and migraine. Rev. Physiol. Biochem. Pharmacol..

[186] Trevisan G., Benemei S., Materazzi S., De Logu F., De Siena G., Fusi C., Fortes Rossato M., Coppi E., Marone I.M., Ferreira J. (2016). TRPA1 mediates trigeminal neuropathic pain in mice downstream of monocytes/macrophages and oxidative stress. Brain.

[187] Materazzi S., Fusi C., Benemei S., Pedretti P., Patacchini R., Nilius B., Prenen J., Creminon C., Geppetti P., Nassini R. (2012). TRPA1 and TRPV4 mediate paclitaxel-induced peripheral neuropathy in mice via a glutathione-sensitive mechanism. Pflugers Arch..

[188] Nakagawa T., Kaneko S. (2017). Roles of Transient Receptor Potential Ankyrin 1 in Oxaliplatin-Induced Peripheral Neuropathy. Biol. Pharm. Bull..

[189] Miyake T., Nakamura S., Meng Z., Hamano S., Inoue K., Numata T., Takahashi N., Nagayasu K., Shirakawa H., Mori Y. (2017). Distinct Mechanism of Cysteine Oxidation-Dependent Activation and Cold Sensitization of Human Transient Receptor Potential Ankyrin 1 Channel by High and Low Oxaliplatin. Front. Physiol..

[190] Trevisan G., Materazzi S., Fusi C., Altomare A., Aldini G., Lodovici M., Patacchini R., Geppetti P., Nassini R. (2013). Novel therapeutic strategy to prevent chemotherapy-induced persistent sensory neuropathy by TRPA1 blockade. Cancer Res..

[191] Miao F., Wang R., Cui G., Li X., Wang T., Li X. (2019). Engagement of MicroRNA-155 in Exaggerated Oxidative Stress Signal and TRPA1 in the Dorsal Horn of the Spinal Cord and Neuropathic Pain During Chemotherapeutic Oxaliplatin. Neurotox. Res..

[192] Sagalajev B., Wei H., Chen Z., Albayrak I., Koivisto A., Pertovaara A. (2018). Oxidative Stress in the Amygdala Contributes to Neuropathic Pain. Neuroscience.

[193] De Logu F., Nassini R., Materazzi S., Carvalho Gonçalves M., Nosi D., Rossi Degl’Innocenti D., Marone I.M., Ferreira J., Li Puma S., Benemei S. (2017). Schwann cell TRPA1 mediates neuroinflammation that sustains macrophage-dependent neuropathic pain in mice. Nat. Commun..

[194] Horváth Á., Tékus V., Bencze N., Szentes N., Scheich B., Bölcskei K., Szőke É., Mócsai A., Tóth-Sarudy É., Mátyus P. (2018). Analgesic effects of the novel semicarbazide-sensitive amine oxidase inhibitor SZV 1287 in mouse pain models with neuropathic mechanisms: Involvement of transient receptor potential vanilloid 1 and ankyrin 1 receptors. Pharmacol. Res..

[195] Chung K.F., Canning B., McGarvey L. (2015). Eight International London Cough Symposium 2014: Cough hypersensitivity syndrome as the basis for chronic cough. Pulm. Pharmacol. Ther..

[196] Kaneko Y., Szallasi A. (2014). Transient receptor potential (TRP) channels: A clinical perspective. Br. J. Pharmacol..

[197] ClinicalTrials.gov. https://clinicaltrials.gov/ct2/show/NCT02432664.

[198] Eberhardt M.J., Schillers F., Eberhardt E.M., Risser L., La Roche J.D., Herzog C., Echtermeyer F., Leffler A. (2017). Reactive metabolites of acetaminophen activate and sensitize the capsaicin receptor TRPV. Sci. Rep..

[199] Schwarz M.G., Namer B., Reeh P.W., Fischer M.J.M. (2017). TRPA1 and TRPV1 antagonists do not inhibit human acidosis-induced pain. J. Pain.

